# Brucellosis in pregnant women from Pakistan: an observational study

**DOI:** 10.1186/s12879-016-1799-1

**Published:** 2016-09-02

**Authors:** Shahzad Ali, Shamim Akhter, Heinrich Neubauer, André Scherag, Miriam Kesselmeier, Falk Melzer, Iahtasham Khan, Hosny El-Adawy, Asima Azam, Saima Qadeer, Qurban Ali

**Affiliations:** 1 University of Veterinary and Animal Sciences, Lahore, Pakistan; 2 Pir Mehr Ali Shah Arid Agriculture University, Rawalpindi, Pakistan; 3 Friedrich-Loeffler-Institut, Institute of Bacterial Infections and Zoonoses, Naumburger Str. 96a, 07743 Jena, Germany; 4 Clinical Epidemiology, Integrated Research and Treatment Center, Center for Sepsis Control and Care (CSCC), Jena University Hospital, Jena, Germany; 5 National Veterinary Laboratories, Islamabad, Pakistan

**Keywords:** Brucellosis, Pregnant women, Pakistan, Abortion, Serology

## Abstract

**Background:**

*Brucella* species occasionally cause spontaneous human abortion. Brucella can be transmitted commonly through the ingestion of raw milk or milk products. The objective of this study was to determine the sero-prevalence of and to identify potential risk factors for brucellosis in pregnant women from Rawalpindi, Pakistan.

**Methods:**

We conducted a cross-sectional study at the Gynecology Outdoor Patient department of the Benazir Bhutto Hospital, Rawalpindi, Pakistan from March to June 2013. Data related to potential risk factors and clinical history was collected by individual interviews on the blood sampling day. The 429 serum samples collected were initially screened by Rose Bengal Plate Agglutination test for the detection of *Brucella* antibodies. We applied standard descriptive statistics and logistic regression analyses.

**Results:**

Twenty five (5.8 %; 95 % confidence interval (CI): 3.8 % -8.5 %) serum samples were found to be seropositive. Brucellosis-related clinical symptoms were recorded in various seropositive cases. Animal contact, raw milk consumption, having an abortion history and the experience of an intrauterine fetal death were associated with seropositivity for brucellosis in univariate analyses (all *p* <0.05). In multiple logistic regression models only the contact with animals remained as independent and robust risk factor (odds ratio 5.21; 95 % CI: 1.88-13.75; *p* = 0.001) for seropositivity.

**Conclusion:**

Brucellosis is a serious threat for pregnant women and their unborn children in Pakistan. Pregnant women having brucellosis-related symptoms or previous history of abortions, miscarriages, intrauterine fetal death and other brucellosis-related manifestations should be screened for brucellosis – especially those exposed to animals given the increased risk – and medication should be administered according to state of the art.

## Background

Human brucellosis is a serious, life threatening disease. Four species of the *Brucella* (*B*.) genus (*B. abortus*, *B. melitensis*, *B. suis* and *B. canis*) are causative agents of brucellosis in humans [1]. Brucellosis is transmitted to humans by direct contact with *Brucella*-infected animals or consumption of their products [2, 3]. Milkers, livestock farmers, abattoir workers, shepherds, veterinarians, meat processing workers and laboratory workers are at high risk of getting infected [4–7].

Brucellosis during pregnancy was first reported in 1908 when Malta fever, i.e. brucellosis, was clinically described [8]. Later on, spontaneous abortions in pregnant women could be associated with the isolation of brucellae from placenta and aborted fetuses [9, 10]. In endemic areas, 1.3 to 12.2 % of pregnant women were found to be seropositive for brucellosis and active brucellosis may be associated with a high abortion rate [11–13]. Complications recorded are abortion, premature delivery, contagious or neonatal brucellosis, intrauterine infection or intrauterine fetal death. *Brucella*-positive infants may develop aspiration pneumonia and meningitis [14–18]. While brucellosis in farm animals is well controlled in most developed countries, it still exists in the Middle East, South and Central America, Africa, the Caribbean, the Mediterranean Basin and in Asia. In Pakistan, brucellosis is endemic in bovines but only a few studies addressed human brucellosis-related risk factors and clinical outcome so far. Recently, 6.9 % of high risk professionals were found to be seropositive. Occupation, locality and consumption of raw milk were identified as associated risk factors. Symptoms were fever, sweating, joint pain, back pain, body pain, abdominal pain, fatigue, lack of appetite and chills [2, 19–21]. Regarding pregnancy-related brucellosis, only a few studies have been conducted world-wide to date [13, 17, 22, 23]. No data are available for Pakistan.

The objective of the present cross-sectional study was to determine if anti-*Brucella* antibodies can be detected in pregnant women from Pakistan at all. Moreover, we were interested in identifying potential risk factors, clinical outcome and symptoms that are associated with a positive serological result.

## Methods

### Study design, setting, participants and data collection

This study has a cross-sectional study design and no follow-up investigation was planned. Blood samples from pregnant women who visited the Gynecology Outdoor Patient (OPD) department from the Benazir Bhutto Hospital, Rawalpindi, Pakistan for routine follow-up controls and who were willing to participate in the study were collected from March to June 2013. No data for those patients who were not willing to participate was recorded at all to guarantee privacy. Data related to potential risk factors for brucellosis, i.e., age, urbanicity, socio-economic status, contact with animals, consumption of raw milk, abortion in household animals, abortion history, contact with women who had aborted, intrauterine fetal death and number of pregnancies before the study, were collected by individual interviews at the time of blood sampling. It was presumed that these patients could not give the exact exposure time because of common ignorance that zoonotic brucellosis does exist in Pakistan. Thus, self-reported clinical symptoms including headache, fever, sweating, muscle or joint pain, joint swelling, general body malaise or backache, fatigue, lack of appetite and mental inattention or depression were recorded for the time interval between pregnancy start and individual interview date for Rose Bengal plate test (RBPT) participants only. An overview of the participant characteristics is summarized in Table 1.

**Table 1:** Characteristics of the 429 Rose Bengal Plate Agglutination test (RBPT) tested pregnant women from Pakistan

Characteristic [unit]	RBPT tested (%)^a^ n _total_ = 429	RBPT positive (%)^a^ n _total_ = 25
Age [Years]		
Median (Q1;Q3)^b^	26 (22;32)	27 (25;36)
18-25	211 (49.18)	11 (44.00)
26-33	118 (27.51)	7 (28.00)
34-41	72 (16.78)	2 (8.00)
>41	28 (6.53)	5 (20.00)
City^c^		
Urban	255 (59.44)	11 (44.00)
Rural	174 (40.56)	14 (56.00)
Socio-economic status^d^		
Low	260 (60.61)	17 (68.00)
Medium	169 (39.39)	8 (32.00)
Contact with animal		
No	389 (90.68)	16 (64.00)
Yes	40 (13.99)	9 (36.00)
Raw milk intake		
No	412 (96.04)	12 (48.00)
Yes	17 (3.96)	13 (52.00)
Contact with abortion in animals		
No	421 (98.14)	24 (96.00)
Yes	8 (1.86)	1 (4.00)
Contact with person with abortion		
No	410 (95.57)	22 (88.00)
Yes	19 (4.43)	3 (12.00)
Number of pregnancies		
Median (Q1;Q3)^b^	2 (1;3)	2 (2;4)
1	150 (35.05)	5 (20.00)
2	106 (24.77)	9 (36.00)
>2	172 (40.19)	11 (44.00)
Abortion history		
No	388 (90.44)	19 (76.00)
Yes	41 (9.56)	6 (24.00)
Intrauterine fetal death		
No	414 (96.50)	22 (88.00)
Yes	15 (3.50)	3 (12.00)
Symptoms^e^		
Headache	ND	13 (52.00)
Fever	ND	12 (48.00)
Sweating	ND	21 (84.00)
Muscle or joint pain	ND	14 (56.00)
Joint swelling	ND	2 (8.00)
General body malaise or backache	ND	23 (92.00)
Fatigue	ND	21 (84.00)
Lack of appetite	ND	7 (28.00)
Mental inattention or depression	ND	9 (36.00)

^a^for “Age” and “Number of pregnancies”, median and interquartile ranges are also reported

^b^(Q1;Q3) is the interquartile range

^c^defined as self-reported location of residence; “rural” means that participants report to live in villages and “urban” means that they report to live in cities

^d^defined as self-reported economic and social position in relation to others

^e^symptoms were assessed in the 25 RBPT positive women only; multiple symptoms can be reported for each woman

### Serology

5 ml blood was collected aseptically into Ethylene Diamine Tetra Acetic acid (EDTA) coated vacutainers. These blood samples were transferred to the National Veterinary Laboratories in Islamabad, Pakistan, in an ice box. For the separation of serum, blood was centrifuged at 5,000 rpm for 15 min and serum was collected in 1.5 ml Eppendorf tubes. Serum samples were then subjected to Rose Bengal plate test (RBPT) [24]. RBPT antigen was purchased from Institute Pourquier, France. Equal amounts of serum and antigen (30 μL) were mixed on a glass plate. The glass plate was agitated for 4 min and the serum was considered positive, if any agglutination occurred. The test was done at the day of admission to the hospital.

### Statistical methods and sample size

Standard descriptive statistics were used to summarize the data (continuous variables: quartiles/count data: absolute and relative frequencies). First, we performed univariate analyses using logistic regression to test for associations between presence/absence of human brucellosis seropositivity and quantitative continuous variables as well as count data. Secondly, we performed multiple logistic regression analyses, including all variables with a univariate *p*-value ≤ 0.2. In case of 5 or less counts in the cross-tables, we report Fisher’s exact test and estimation results only. All reported *p*-values are two-sided and not corrected for multiplicity. In this explorative study, we applied a significance level α = 0.05 (two-sided).

A sample size of 400 participants has a comparison-wise power of 80 % to detect generic exposure-outcome associations for a rate of 5 % exposed participants and odds ratios >2.8 (significance level α = 0.05 (two-sided) for a X^2^ test and a 1:1 distribution of the outcome); for higher rates of e.g. 10 % exposed participants odds ratios >2.2 are detectable with a similar comparison-wise power (proc power of SAS 9.3; SAS Institute Inc., Cary, NC, USA). Thus, the study was well-powered to detect strong associations with presence/absence of human brucellosis seropositivity.

All statistical analyses were done using the statistical language R 3.0.2.

## Results

Four hundred twenty nine pregnant women participated in the study (see Table 1) and 25 (5.8 %; 95 % CI: 3.8 %-8.5 %) were found to be RBPT positive.

All pregnant women reported more than one of the symptoms usually associated with clinical brucellosis. The time of pregnancy did not influence the clinical presentation of disease i.e. there was no correlation found between pregnancy status and number of reported symptoms or self-reported severity of these symptoms. The time of infection or onset of disease could not be elucidated by the design of our questionnaire.

The results of the association analyses concerning presence/absence of human brucellosis seropositivity are given in Table 2. Animal contact (*p* < 0.001), raw milk intake (*p* < 0.001, Fisher’s exact test), abortion history (*p* = 0.016) and the experience of an intrauterine fetal death (*p* = 0.050, Fisher’s exact test) were associated with seropositivity. For all these univariate associations we estimated strong association effects (see Table 2) but the estimation itself was highly imprecise due to the small number of only 25 seropositive women. Additionally, we modeled the impact of the variables jointly and detected that only the contact with animals was a significant, independent and relatively robust predictor of presence/absence of seropositivity (odds ratio 5.21; 95 % CI: 1.88-13.75; *p* = 0.001).

**Table 2:** Results of the logistic regression analyses, Odds ratios (ORs) with 95 % confidence intervals (CIs) and two-sided *p*-values are provided for the univariate and multiple models (Details see text). Note that the left column lists both units (for quantitative variables) or reference values for categorical variables in parenthesis

	Univariate logistic models		Multiple logistic model	
Variable [unit or reference (REF)]	OR (95 % CI)	*p*-value	OR (95 % CI)	*p*-value
Age [per year]	1.05 (0.99, 1.10)	0.093	1.03 (0.97, 1.09)	0.299
Rural city [REF urban]^a^	1.94 (0.86, 4.48)	0.110	1.34 (0.56, 3.24)	0.509
Medium socio-economic status [REF low]^b^	0.71 (0.28, 1.64)	0.437		
Contact with animal [REF no contact]	6.77 (2.67, 16.34)	<0.001	5.21 (1.88, 13.75)	0.001
Raw milk intake [REF no raw milk intake]^c^	102.86 (27.35, 390.43)	<0.001		^d^
Contact with abortion in animals [REF no contact]^c^	2.36 (0.10, 18.69)	0.384		
Contact with person with abortion [REF no contact]^c^	3.29 (0.76, 12.40)	0.091		^d^
Number of pregnancies [per one]	1.16 (0.89, 1.49)	0.265		
Presence of abortion history [REF absence]	3.33 (1.15, 8.47)	0.016	1.57 (0.47, 4.64)	0.439
Experience of intrauterine fetal death [REF no experience]^c^	4.43 (1.00, 16.24)	0.050		^d^

^a^defined as rural mean patients live in village and urban mean patients live in city

^b^defined as patient belong to middle class versus patients of poor families

^c^result of Fisher's exact

^d^not calculated due to too few observations

## Discussion

Brucellosis is an infectious disease which affects both animals and humans. It is endemic in ruminants in Pakistan but little is known on human brucellosis, especially in pregnant women who are considered to be at special risk of abortion.

The various studies on human brucellosis and pregnancy have to be compared with caution due to the different cohorts investigated, techniques used or prevalent epidemiological situation [25]. It is obvious that our study population is not representative for the group of ‘pregnant women in Pakistan’ as it was not random due to restrictions in the acquisition of participants. In a comparable study population 3.5 % of pregnant women from rural areas of Saudi Arabia were seropositive [11]. In the present study, 5.8 % of pregnant Pakistani women were found to be seropositive and indeed women from rural areas were more often seropositive than those from urban areas. These women are often engaged in animal keeping and thus are in close contact to infected animals or abortion material. Thus, our women may be exposed to a similar risk as high risk professionals in Pakistan - a group in which 6.9 % of the persons were recently found to be seropositive [2]. Our findings are corroborated by the data of a survey from Turkey, where 59 % of pregnant women with brucellosis were stockbreeders [16]. 25 % of Rwanda women suffering from abortions and stillbirth were found to be infected with brucellosis. Again, these women were in close contact to cattle, goats and sheep [26].

Pakistani women from rural, low income families were more often seropositive (6.5 %) than those from better situated families of urban areas (4.7 %). Pregnant women with an abortion history, with contact to animals that aborted or with contacts to women who had an abortion were more often seropositive (14.6 %, 12.5 % and 15.8 %, respectively), although the latter two factors were not significantly associated with seropositivity in our study. These findings may jointly indicate that brucellosis has already become chronic in our women and may have caused multiple abortions. Alternatively, reinfections might have occurred frequently given that their living conditions were not altered. It may be speculated that these women simply had no adequate access to medical care due to poor economic situation as all these women reported symptoms of active brucellosis. Thus, brucellosis proved again to be a disease sustained by poverty. Childlessness caused by (chronic) brucellosis may have also negative impact on the social standing of a family in the society or for security in old age. Especially the mental pressure on the childless woman may be high. Future studies should reflect on these issues, too.

Pregnant women consuming raw milk were more often seropositive (76.5 %) compared to those never consuming raw milk (2.9 %). However, the overall rate of reported raw milk consumption was relatively low (~4 %). Nevertheless, raw milk consumption-related high-frequent *Brucella* seropositivity was also observed in pregnant women from Rwanda and Turkey [16, 25, 26]. The lack of knowledge about the presence of brucellosis in livestock, its transmissibility and adequate countermeasures e.g. heating of milk, or indifference fosters human brucellosis. Programs to educate illiterate agricultural population are needed in Pakistan to reduce disease incidence. Efficient regulations on food hygiene should be placed into force.

However, brucellosis in pregnant women poses also severe risks for newborns. Infection was shown to be a leading cause of neonatal mortality among infants delivered at a tertiary hospital in Karachi, Pakistan [27]. Congenital transmission of brucellosis was reported from Argentina [18, 28] in an infant whose parents were livestock farmers. Transmission by breast milk has already been described [29–31]. Now, future studies are needed to determine the share of brucellosis on neonatal mortality in Pakistan.

Our study has several limitations. First, while the Rose Bengal plate test (RBPT) is a feasible and reliable test especially as screening test in our study setting, confirmation should be done using other tests (e.g. Complement Fixation Test or Enzyme Linked Immunosorbent Assay). Secondly, the number of 25 RBPT positive women results in highly imprecise odds ratio estimators. Thirdly, a major drawback of our study is that the prevalent *Brucella* species remains unknown and molecular epidemiology cannot be used to identify chains of infection. Use of genus or species specific quantitative real-time polymerase chain reaction (qRT PCR) and molecular typing directly from the sample will help to overcome these weaknesses. For the moment we cannot exclude that a relevant number of cases might have been caused by veterinary vaccine which still have a considerable pathogenicity for humans. Handling these vaccines or freshly vaccinated animals still excreting the vaccine strain will pose a special risk for pregnant women in rural areas. Both, vaccination and awareness for the pathogenicity of vaccines have to be objects of future questionnaires.

We admit that the data obtained have only restricted significance. However, they might convince public health policy makers, physicians and families having religious reservations to contribute to future studies investigating the risks of brucellosis in pregnancy. Thus, counterprograms for veterinary and human public health can be tailored and implemented in Pakistan.

## Conclusions

Brucellosis is considered to be the most common zoonotic infection worldwide and a serious hazard for pregnant women. Humans with brucellosis-related symptoms or manifestations (including a history of abortions) should be investigated for brucellosis; subsequent care should be according to the state of the art. Rose Bengal plate test (RBPT) should be considered as a screening test that must be confirmed by other reliable tests. This study confirmed that brucellosis is a serious threat and public health concern for pregnant women and their unborn children and more generally for humans in contact with animals or raw milk consumption in Pakistan. The findings reinforce the need for adequate preventive and control measures to be taken against zoonotic brucellosis especially in one of the most susceptible and vulnerable individuals of the human population.
